# A Quantitative Evaluation of Hepatic Uptake on I-131 Whole-Body Scintigraphy for Postablative Therapy of Thyroid Carcinoma

**DOI:** 10.1097/MD.0000000000001191

**Published:** 2015-07-17

**Authors:** Michihiro Nakayama, Atsutaka Okizaki, Miki Sakaguchi, Shunta Ishitoya, Takahiro Uno, Junichi Sato, Koji Takahashi

**Affiliations:** From the Department of Radiology (MN, AO, SI, KT), Asahikawa Medical University; and Division of Radiology (MS, TU, JS), Asahikawa Medical University Hospital, Asahikawa, Japan.

## Abstract

This study aimed to determine clinical association between quantitative hepatic uptake on postablative whole-body scan (WBS) with differentiated thyroid cancer (DTC) prognosis.

We analyzed 541 scans of 216 DTC patients who were divided into 3 groups based on radioactive iodine (I-131) WBS uptake and clinical follow-up: group 1 (completion of ablation), group 2 (abnormal uptake in the cervical region), and group 3 (abnormal uptake with distant metastases). For each group, we calculated the ratio of I-131 WBS hepatic uptake (H) to cranial uptake as background (B); this ratio was defined as H/B. Furthermore, we made a distinction between group 1, as having completed radioactive iodine therapy (RIT) (CR), and group 2 and 3, as requiring subsequent RIT (RR).

The average H/B scores were 1.34 (median, 1.36; range 1.00–2.1) for group1; 1.89 (median, 1.75; range 1.41–4.20) for group 2; and 2.09 (median, 1.90; range 1.50–4.32) for group 3. Bonferroni multiple comparisons revealed significant differences in H/B among these groups. The H/B of group 1 was significantly smaller than that of other 2 groups (*P* < 0.0001). The precise cutoff value of H/B for therapeutic effect was ≤1.5. Moreover, 159 of 160 scans in the CR and 375 of 381 patients in the RR were correctly diagnosed using this cutoff value in the final outcome of RIT, yielding a sensitivity, specificity, positive predictive value, and negative predictive value of 99.4%, 98.4%, 99.7%, and 96.3%, respectively.

Increased hepatic uptake of I-131 on WBS may predict disease-related progression.

## INTRODUCTION

An estimated 62,980 new cases of thyroid cancer were expected to be diagnosed in 2014 in the United States, with 3 in 4 cases occurring in women. The incidence rate of thyroid cancer has been sharply increasing since the mid-1990s in both men and women.^1^ From 2005 to 2009, there was an increase in annual incidence rates by 5.6% in men and 7.0% in women per year. Moreover, an estimated 1890 deaths from thyroid cancer were expected in 2014 in the United States. From 2005 to 2009, the death rate for thyroid cancer was stable at 0.5 per 100,000 in both men and women.^1^ The initial treatment for majority of patients with differentiated thyroid cancer (DTC) is total thyroidectomy.^2^ Radioactive iodine (I-131) therapy (RIT) is an effective treatment for relapse or metastasis after DTC surgery and significantly improves the prognosis of recurrent DTC.^3^

Currently, response to RIT is evaluated by thyroglobulin concentration in addition to findings with conventional imaging, such as ultrasound, x-ray, computed tomography (CT), and I-131 scintigraphy.^4,5^ Post-RIT whole-body scans (WBSs) frequently reveal diffuse I-131 uptake in the liver^6–10^; this diffuse pattern does not represent liver metastases that usually appear as discrete lesions.^11^ Organoiodine compounds are metabolized by the liver; therefore, hepatic uptake on post-RIT scan suggests the presence of organic iodine.^12–15^ Some authors considered diffuse hepatic uptake to be a sign of treatment benefit^7,9,11,16–18^; however, others reported it to be a nonspecific finding^6,8,9^ It is unclear whether the iodinated proteins observed are derived solely from thyroid tissue. Therefore, this study aimed to investigate whether the quantitative evaluation of hepatic uptake is associated with disease progression in DTC patients.

## MATERIALS AND METHODS

### Study Population

This was a retrospective review. Data were collected from the records of consecutive DTC patients at our hospital between April 2004 and January 2014. Exclusion criteria were as follows: missing biochemical and/or imaging parameters, presence of liver metastases, and/or abnormal liver function and/or follow-up of <6 months. All patients had primarily undergone total thyroidectomy and received I-131 ablation with a mean activity of 5.3 GBq (range 3.70–5.55 GBq) after the withdrawal of hormone therapy for at least 2 weeks. After the RIT, every patient was taken care of as an outpatient.

**FIGURE 1:**
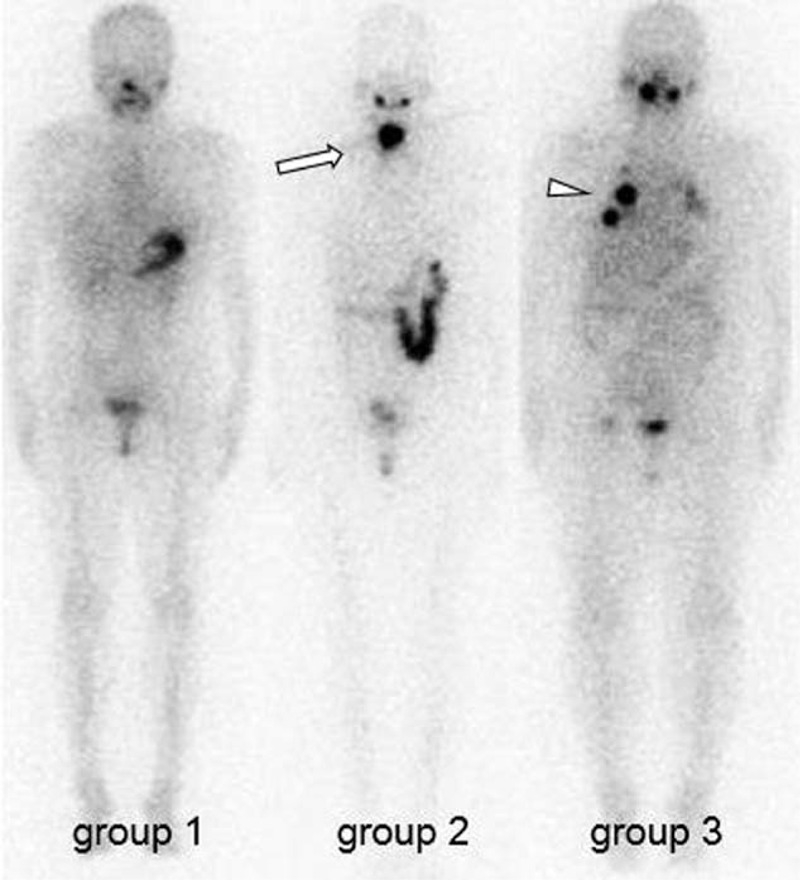
Representative I-131 WBS anterior view images of patients in the 3 groups who were followed up after I-131 RIT for DTC. *Arrow* = cervical abnormal uptake. *Arrow head* = metastatic uptake. All other areas of I-131 uptake were physiologic (salivary glands, intestines, and bladder). DTC = differentiated thyroid cancer, I-131 = radioactive iodine, RIT = radioactive iodine therapy, WBS = whole-body scan.

The study patients were classified into 3 groups by the visual assessment of I-131 WBS: group 1 = completion of ablation; group 2 = abnormal uptake of cervical region, including thyroid bed; and group 3 = that of metastasis, as independently rated by 2 experienced nuclear physicians. Representative cases are shown in Figure 1. A patient was considered to have completed ablation if thyroglobulin did not increase for at least 6 months of follow-up and if there were no imaging studies or clinical findings consistent with persistent or recurrent disease. Furthermore, we made a distinction between the groups by defining group 1 as those who completed RIT (CR) and groups 2 and 3 as those who required subsequent RIT (RR).

### Image Analysis

WBS was performed 4 days after I-131 administration. An anterior planar projection was acquired using dual-head gamma cameras (Millennium VG, GE Medical System, Tokyo, Japan) equipped with high-energy medium-sensitivity collimators. Scan velocity was 15 cm/min. A matrix size of 256 × 1024 pixels and a symmetric window of 20% centered on a 364 keV photopeak were used for all acquisitions.

In the WBS anterior view, region of interests (ROIs) were set on the liver and on the cranial region to minimize individual variations caused by bone marrow and soft tissue on the background. The hepatic uptake ratio (H/B) was calculated using the following formula: H/B = (maximum hepatic uptake counts)/(maximum background counts). ROIs for the liver and background were defined manually by 2 experienced nuclear physicians and 1 radiology technician on the basis of a visual boundary. H/B was evaluated in blinded fashion and was calculated as the average of each value determined by those 3 experts. CT images were reviewed to facilitate ROI determination.

### Statistical Analysis

Data analysis was conducted using statistical software (XLSTAT2014, Addinsoft, Paris, France). Measurements for the same lesion from these 3 readers were averaged and the mean values were used for further analyses. Differences in H/B were assessed by the Kruskal–Wallis test. Bonferroni multiple comparison was used to identify groups that were different from the others.

Differences in H/B between CR and RR were analyzed using the Wilcoxon signed-rank test. Receiver operating characteristic (ROC) curve was derived using the H/B of CR and RR. The sensitivity, specificity, and positive and negative predictive values were determined from the optimal cutoff values using the ROC curve. *P* values <0.05 were considered statistically significant.

### Ethics

Informed consent on secondary use of clinical information for research was obtained from all patients who participated in the study. This study was retrospective, and the data were analyzed anonymously; therefore, ethics committee approval was deemed unnecessary at our institution.

## RESULTS

### Patient Characteristics

During the analysis period, a total of 615 WBS were performed after treatment with I-131 for DTC. In this study, 541 scans of 216 patients were included. Demographic data, histological types, and tumor-node-metastasis (TNM) classification are shown in Table 1. Pathological classification of thyroid tumors was according to the TNM version 7 (2009).^19^

**TABLE 1:**
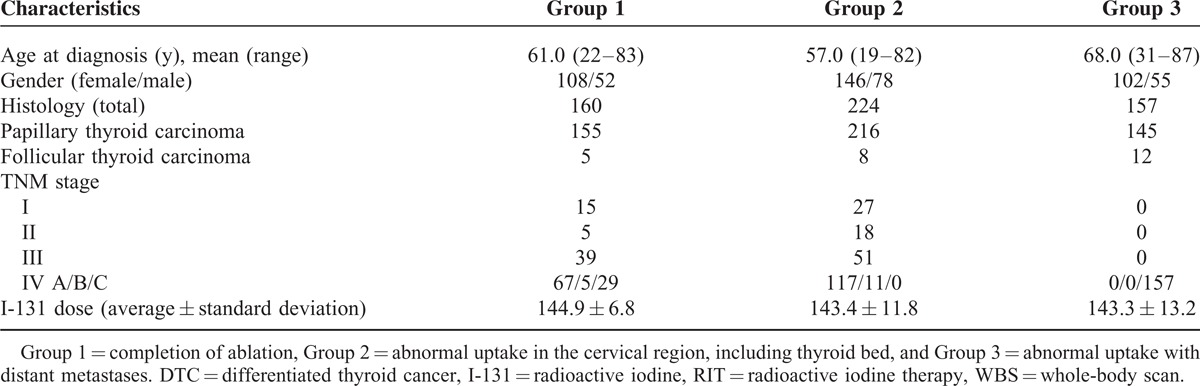
Characteristics of Patients in Each Group Who Underwent I-131 WBS After RIT for DTC (N = 541)

### Hepatic Uptake Ratio and Clinical Outcome

**FIGURE 2:**
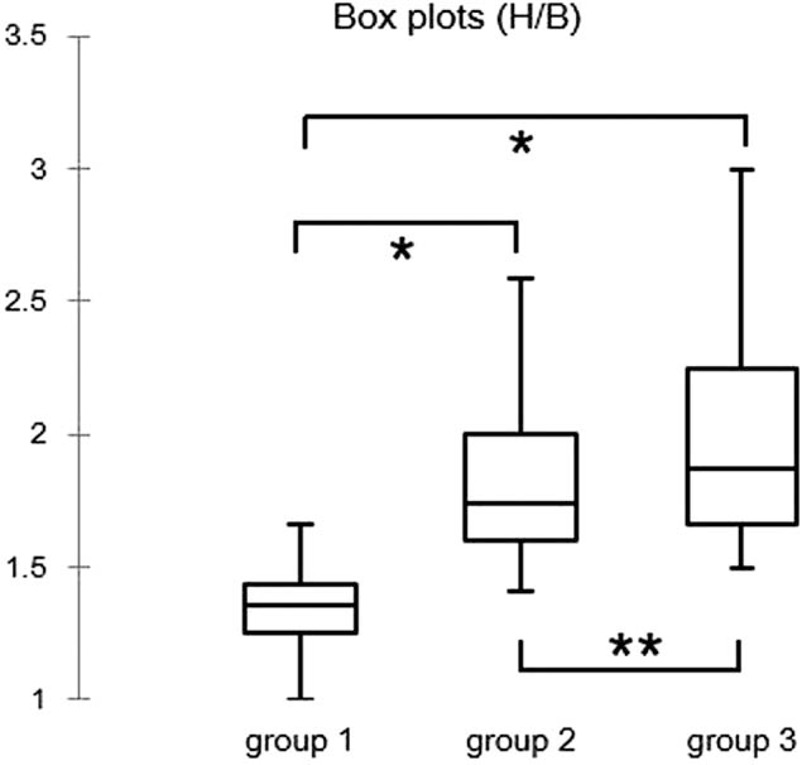
Box plots showing the distribution of groups 1, 2, and 3 by Bonferroni multiple comparison. ^∗^*P* < 0.0001, compared with group 1; ^∗∗^*P* < 0.0003, compared with group 2.

**FIGURE 3:**
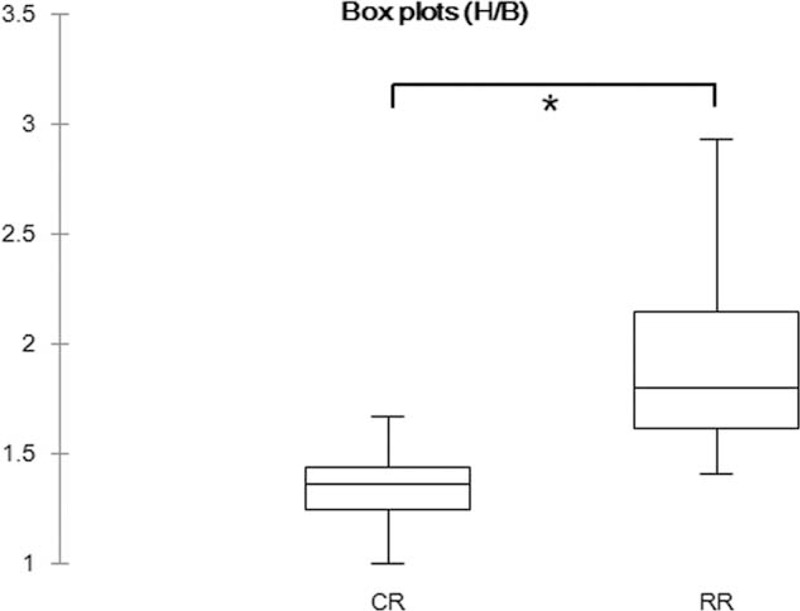
Box plots showing the distribution of CR and RR by Wilcoxon signed-rank test. ^∗^*P* < 0.0001. CR = completed radioactive iodine therapy, RR = required subsequent radioactive iodine therapy.

The average H/B scores were 1.34 (median, 1.36; range 1.00–2.1) for group1; 1.89 (median, 1.75; range 1.41–4.20) for group 2; and 2.09 (median, 1.90; range 1.50–4.32) for group 3. There were significant differences in H/B among these 3 groups (*P* < 0.0001 and *P* < 0.00003). The H/B of group 2 was significantly greater than that of the other 2 groups (*P* < 0.00001); the average H/B score of RR was 1.97 (median, 1.80; range 1.41–4.32), and that of CR was the same as that of group 1. There was a significant difference between CR and RR (*P* < 0.00001). The box plots are shown in Figure 2 and Figure 3.

### ROC Analysis

**FIGURE 4:**
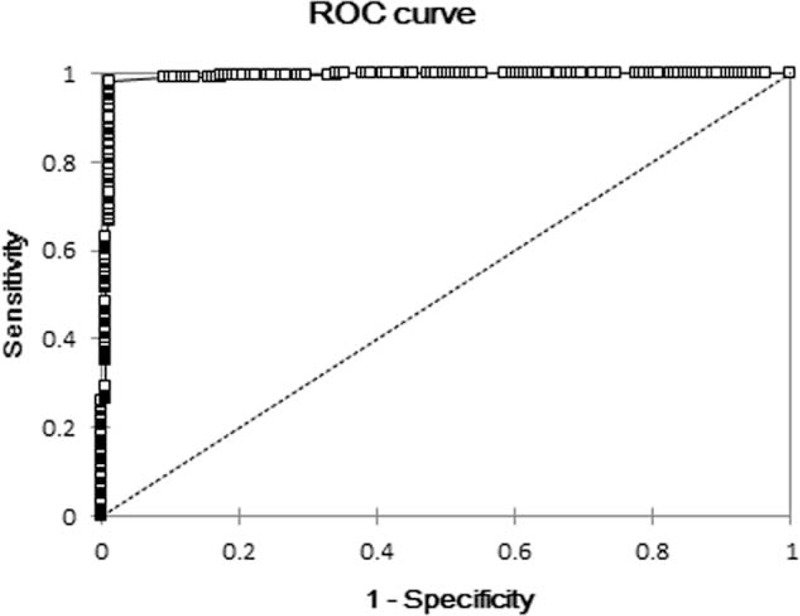
ROC curve analysis. For DTC patients undergoing WBS after I-131 RIT, H/B differentiated between CR and RR and the precise cutoff value was 1.5. CR = completed radioactive iodine therapy, DTC = differentiated thyroid cancer, H/B = hepatic uptake ratio, I-131 = radioactive iodine, RIT = radioactive iodine therapy, ROC = receiver operating characteristic, RR = required subsequent radioactive iodine therapy, WBS = whole-body scan.

Detailed results from the final outcome of RIT are presented in Table 2. The precise cutoff value of H/B for therapeutic effect was ≤1.5 (Figure 4). Moreover, 159 of 160 scans in the CR and 375 of 381 patients in the RR were correctly diagnosed using this cutoff value, yielding a sensitivity of 99.4%, specificity of 98.4%, positive predictive value of 96.4%, and negative predictive value of 99.7%. The area under the curve of H/B was 0.991.

**TABLE 2:**
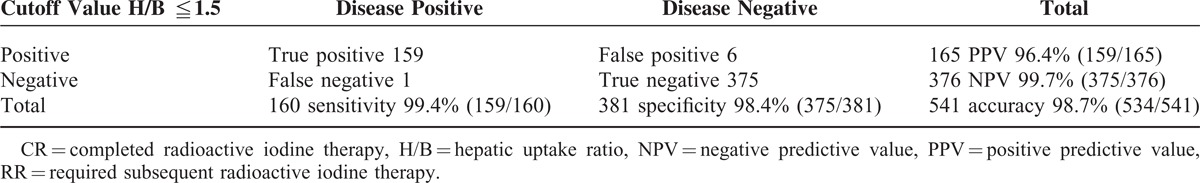
Diagnostic Values for Differentiation Between CR and RR Based on Parameters of H/B

## DISCUSSION

DTC patients are evaluated by chest radiography, ultrasound, CT, I-131 WBS, and thyroglobulin concentration after total thyroidectomy. Post-RIT WBS provides the physician with important information, including the presence of metastatic disease and iodine avidity of residual thyroid tissue.^20^ As an extremely useful marker of metastasis and relapse, the concentration of serum thyroglobulin, which is synthesized by thyroid follicular cells, is widely used for tumor evaluation after total thyroidectomy for DTC. However, coexistent serum thyroglobulin autoantibodies (TgAb), which were reported in 7.5% to 25% of DTC patients,^21–26^ can underestimate thyroglobulin measurement by immunometric assays.^27–30^ Furthermore, Albert and Puliafito^31^ reported false-positive results of thyroglobulin tests. H/B may have limited clinical significance, but might be found to be clinically valuable when the patients have TgAb.

Iodine does not normally concentrate in the liver.^19^ The majority of thyroid hormones in the thyroid gland and plasma are levothyroxine. Most levothyroxine is converted to triiodothyronine,^32^ a more metabolically more active form, by deiodination in liver, skeletal muscle, kidney, brain, and other tissues, whereas the rest is conjugated with sulfate and glucuronide in the liver, excreted in bile, and partially hydrolyzed in the bowel. This could be a possible reason that diffuse hepatic uptake of radioiodine is frequently observed in WBS.^33^ Studies of physiologic radioiodine uptake in the liver are shown in Table 3. Some authors have reported diffuse hepatic uptake on I-131 WBSs.^9,10,16,17,33–36^ Chung et al^16^ found that I-131-labeled thyroglobulin was related to hepatic uptake, and that hepatic uptake indicated functioning thyroid remnant or metastasis. In patients without thyroid remnant, radioiodinated thyroglobulin released from functioning cancer tissue is regarded as the cause of diffuse hepatic uptake of radioiodine. These authors suggested that this finding is also evidence of association between diffuse hepatic uptake and the presence of thyroid remnants, metastatic DTC lesions, which is in agreement with our current hypothesis. Furthermore, Jun et al^9^ reported that the diffuse hepatic uptake intensity reflects the amount of destroyed thyroid tissue or functioning metastasis. However, other investigators have stated that diffuse hepatic uptake is a benign finding without clinical importance.^6,8,10^ A more recent study conducted by Lee et al^10^ revealed no correlation of hepatic uptake with thyroid remnant, and presence of distant metastatic foci. However, by visual score, the criteria used to determine the presence of hepatic uptake often depended on the physicians’ personal experience; therefore, the results are variable. Some studies indicated a correlation between liver uptake and I-131 dose^16,17,37^; however, no relationship with the dose administered to each group was observed in our study. However, H/B may have an advantage of not being dependent on dose and test date because it can normalize hepatic uptake by background.

**TABLE 3:**
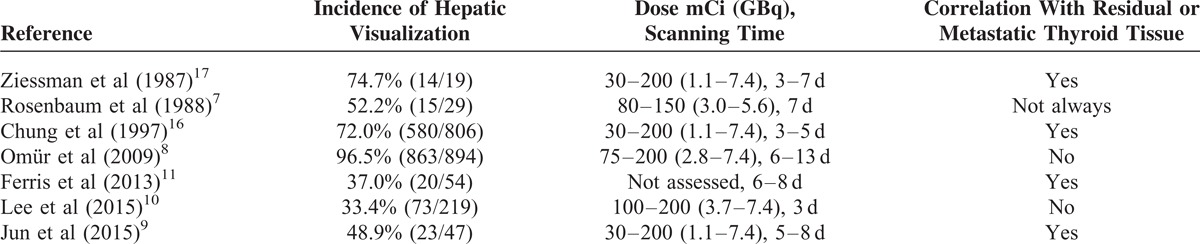
Review of Diffuse Hepatic Uptake of Radioiodine

In this study, we quantitatively evaluated hepatic uptake on I-131 WBS after RIT. H/B was associated with disease progression; moreover, if H/B fell below a certain level, DTC patients may have an extremely low risk for cancer recurrence. The area under the curve of H/B was 0.991 that led to high sensitivity and specificity. H/B and residual thyroid tissue or metastases were found to be associated, and this was likely the mechanism for diffuse hepatic uptake.

Maximum hepatic uptake was positively correlated with both liver volume and radioactivity per unit volume.^38,39^ Furthermore, the maximum value would probably not be affected by the digestive tract even if ROI settings were incomplete because I-131 intrahepatic distribution was relatively uniform.^12,17,31^

This study had several limitations. First, selection bias is inevitable because the present study is a retrospective single-center study. Second, the follow-up period of our study was relatively short. Third, because it was a single-center study, the number of subjects was relatively small, despite the fact that the data were collected from a 10-year period. As our institution is still performing RIT, a study with more patients and longer follow-up period can still be carried out in the future. Fourth, the occasional high uptakes from perspiration and/or intestinal tracts rendered difficult quantitative evaluations, necessitating patients to take a shower and/or use laxatives before the scan. Fifth, the cutoff value was based on data collected at our institution alone, and the value of H/B may have varied according to the imaging system used. We intend to address these issues by conducting similar comparisons at multiple institutions. Finally, in general, this assessment was performed three-dimensionally; therefore, the effects of absorptive scattering correction and liver morphology may have been ignored. However, even if such errors were overlooked, it appears to be an extremely easy to use index with sufficiently demonstrated results. In future, we plan to investigate on a hepatic uptake method that can be used for a more accurate quantitative evaluation.

## CONCLUSIONS

Increased hepatic uptake on I-131 WBS may predict disease progression. In clinical practice, patients are usually taken off current treatment if they have disease progression/recurrence. When patients who received RIT were considered disease progression, they may select other treatment such as molecular targeted therapy. Thyroglobulin concentration cannot be used to determine treatment response in patients who are TgAb positive. In such a case, H/B might be presented to help us determine whether disease progression was observed or not.
